# Correlative light electron ion microscopy reveals in vivo localisation of bedaquiline in *Mycobacterium tuberculosis*–infected lungs

**DOI:** 10.1371/journal.pbio.3000879

**Published:** 2020-12-31

**Authors:** Antony Fearns, Daniel J. Greenwood, Angela Rodgers, Haibo Jiang, Maximiliano G. Gutierrez

**Affiliations:** 1 Host–Pathogen Interactions in Tuberculosis Laboratory, The Francis Crick Institute, London, United Kingdom; 2 School of Molecular Sciences, University of Western Australia, Perth, Australia; 3 Department of Chemistry, The University of Hong Kong, Hong Kong, China; Washington University in St. Louis, UNITED STATES

## Abstract

Correlative light, electron, and ion microscopy (CLEIM) offers huge potential to track the intracellular fate of antibiotics, with organelle-level resolution. However, a correlative approach that enables subcellular antibiotic visualisation in pathogen-infected tissue is lacking. Here, we developed correlative light, electron, and ion microscopy in tissue (CLEIMiT) and used it to identify the cell type–specific accumulation of an antibiotic in lung lesions of mice infected with *Mycobacterium tuberculosis*. Using CLEIMiT, we found that the anti-tuberculosis (TB) drug bedaquiline (BDQ) is localised not only in foamy macrophages in the lungs during infection but also accumulate in polymorphonuclear (PMN) cells.

## Abstract 2

This study uses correlative light, electron and ion microscopy (CLEIM) in vivo to reveal the intracellular fate of an antibiotic in lung lesions of mice infected with Mycobacterium tuberculosis, with organelle-level resolution.

## Introduction

An effective chemotherapy against bacterial infections must include antibiotics with pharmacokinetic properties that together allow penetration into all infected microenvironments [**1**]. Antimicrobial penetration is especially important for the treatment of infections where antibiotics need to reach intracellular bacteria [2], including *Mycobacterium tuberculosis*. In tuberculosis, treatment requires at least 3 antibiotics for 6 months [3], and we do not fully understand why this extended treatment is needed. In this context, understanding how tissue environments affect antibiotic localisation, exposure, and consequently efficacy against the pathogen is crucial [4].

Although it is critical to define if antimicrobials are able to reach their intracellular targets, imaging of antibiotics (and drugs in general) at the subcellular level in infected tissues remains challenging. Only recently have studies in vivo determined antibiotic distributions in granulomatous lesions by matrix-assisted laser desorption–ionisation mass spectrometric imaging (MALDI-MSI) [5]. However, this approach only allows analysis at the tissue level and does not reach subcellular or even cellular resolution [6]. On the other hand, nanoscale secondary ion mass spectrometry (nanoSIMS) is an analytic technique that can be used to generate images based solely on isotopic content at subcellular resolution in biological systems [7]. NanoSIMS uses focused primary ion beam to scan sample surface and generate secondary ions which are detected by a mass analyser, and it has been used to visualise drugs at 50-nm resolution in cells [8] and tissues [9]. However, there are limitations with this method such as the lack of correlation with other available imaging modalities that provide spatial information of specific cell types localisation and function. Thus, correlative approaches are needed to obtain both spatial localisation of drugs and biologically relevant information from experimental systems [7]. Recently, a correlative imaging approach combining correlative light, electron, and ion microscopy (CLEIM) has been developed for subcellular antibiotic visualisation in vitro cultured cells [10]. However, there are currently no approaches available that allow correlative studies at the subcellular resolution in vivo.

## Results

**Fig 1:**
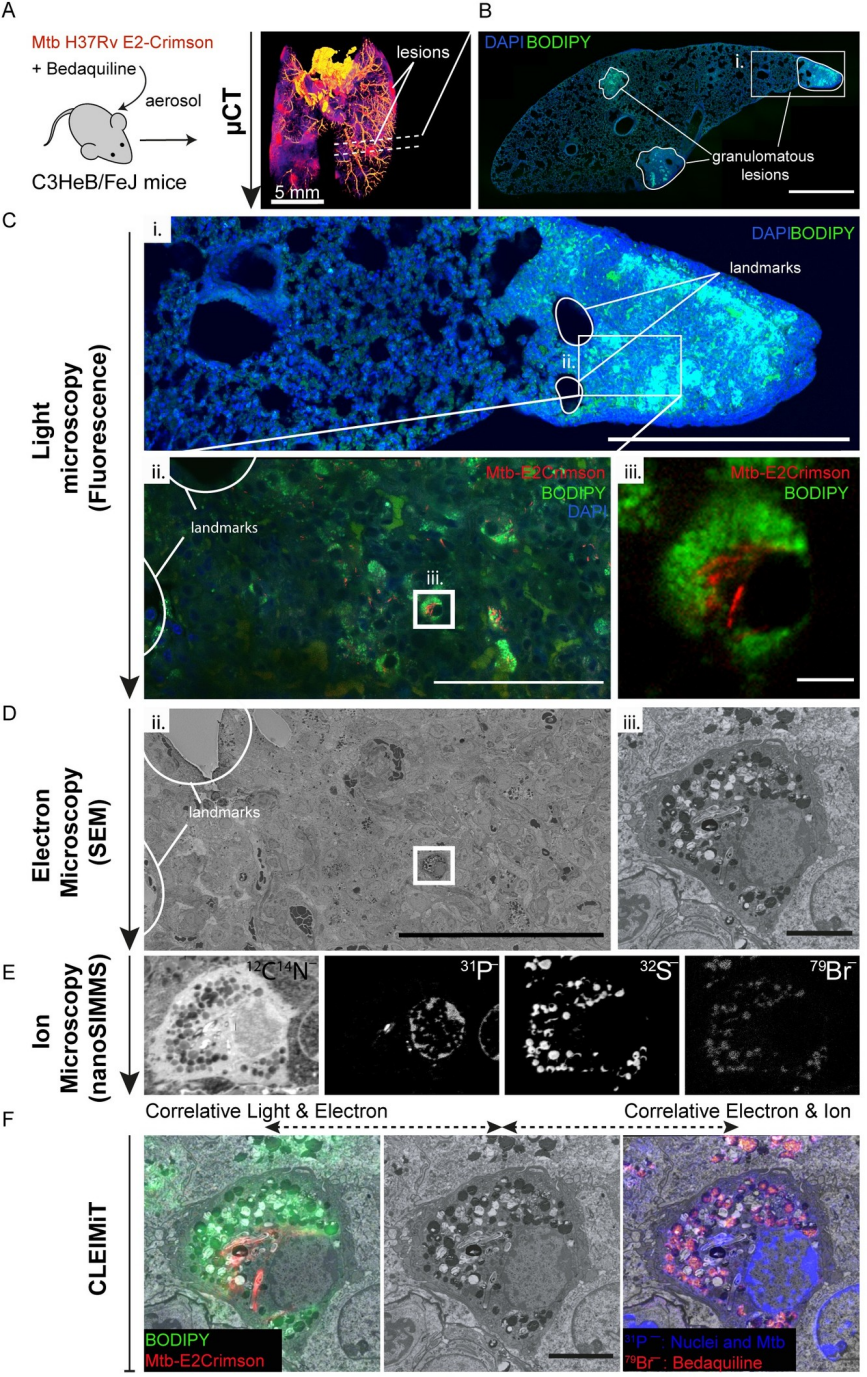
The CLEIMiT workflow and correlative imaging of BDQ in *M*. *tuberculosis*–infected foamy macrophages within lung tissue. (**A**) Diagram illustrating the in vivo experimental setting. C3HeB/FeJ mice were infected with *M*. *tuberculosis* H37Rv expressing E2-Crimson (Mtb-E2Crimson) by aerosol. After 21 days, infected mice were treated with 25 mg/kg of BDQ or vehicle daily for 5 days via oral gavage. Lungs were removed, fixed with 10% formalin, contrasted, and embedded in LMA then imaged by μCT for sequential vibratome sectioning. (**B**) Fluorescence microscopy: a tile scan of a tissue section (approximately 100-μm thickness) stained with DAPI (blue) and BODIPY (green), granulomatous lesions are marked with a solid white line to indicate the boundary (scale bar = 1,000 μm). (**C**) Light microscopy of an ROI: (i) zoomed fluorescence image (white box from Fig 1B), “landmarks” used for downstream location recognition, are indicated by the solid white boundary lines (scale bar = 500 μm). White rectangle shows the ROI for downstream analysis. (ii) A confocal image of the region indicated by the white box above shows an area of strong cellular infiltration and the accumulation of BODIPY (green) positive cells. Cells infected with Mtb-E2Crimson (red) are also visible throughout this region. The same landmarks marked in image (i) are present. White box indicates the selected infected foamy cell. Scale bar = 100 μm. (iii) Zoomed in image showing the selected foamy cell infected with Mtb-E2Crimson (red) for correlative analysis. Scale bar = 5 μm. (**D**) Electron microscopy of the ROI: (ii) tissue overview (600× magnification) with landmarks present, white box indicates the selected infected foamy cell. Scale bar = 100 μm. (iii) Zoomed in image showing the selected foamy cell infected with Mtb (15,000× magnification). Scale bar = 5 μm. (**E**) Ion microscopy of the selected cell: Panel shows the individual nanoSIMS images for the following ion signals from left to right; ^12^C^14^N^–^, ^31^P^–^, ^32^S^–^, and ^79^Br^–^. (**F**) CLEIMiT: Left, a correlated image overlaying fluorescent signal from BODIPY (green) and Mtb-E2Crimson (red) against the SEM image. Right, a correlated image overlaying the ^79^Br^−^and ^31^P^–^ signals with the SEM image. Center, the corresponding SEM image of the infected foamy cell. Scale bar = 5 μm. BDQ, bedaquiline; CLEIMiT, correlative light, electron, and ion microscopy in tissue; LMA, low melting point agarose; nanoSIMS, nanoscale secondary ion mass spectrometry; ROI, region of interest; SEM, scanning electron microscopy; μCT, micro-computed tomography.

With the aim to define the subcellular localisation of antibiotics in infected cells within tissues, we used a mouse model of tuberculosis. Our goal was to develop an imaging approach to analyse the distribution of antibiotics from complex tissues to individual cells at the subcellular level in infected lungs. For that, we infected susceptible C3HeB/FeJ mice with *M*. *tuberculosis* H37Rv expressing fluorescent E2-Crimson via aerosol infection (Fig 1A). The C3HeB/FeJ susceptible mouse strain develops necrotic lesions in the lung that better recapitulate human granulomas, a hallmark of tuberculosis infection [11]. After 21 days of infection, mice were treated daily for 5 days either with control vehicle or 25 mg/kg of the antimycobacterial antibiotic bedaquiline (BDQ). As previously reported [12], this treatment reduced approximately 10-fold the bacterial loads in the lungs, as measured by colony-forming units (CFU) counting (S1A and S1B Fig and S2 Data). Following treatment, mice were humanely killed and fixed by perfusion with formalin. Lungs were removed and granulomatous lesions were visualised by micro-computed tomography (μCT, Fig 1A, S1C Fig and S1 Movie). Replicate lung tissues were embedded in agarose for further processing and imaging.

One of the main technical challenges of our attempt to define if the antibiotic reached intracellular bacteria was to identify and correlate across the different imaging modalities and scales the infected cells present in the lung. We devised a strategy that included the identification of a granulomatous lesion within 100-μm thickness sections and nondestructive 3D imaging by confocal laser scanning microscopy of the entire section as well as the region of interest (ROI; Fig 1B and 1C). Vibratome sections were stained with DAPI to visualise nuclei and BODIPY 493/503 to visualise lipid droplets (LDs), previously shown to accumulate in foamy macrophages in necrotic lesions [13]. In agreement with previous studies, we found that granulomatous lesions were heavily enriched in LD-laden foamy macrophages (Fig 1B and 1C). After fluorescence imaging, sections were recovered, and resin embedded for electron microscopy. In order to correlate the 3D fluorescence microscopy with the electron microscopy, sections were analysed by μCT 3D scanning (S2 Fig). This approach enabled the precise localisation of the ROI previously imaged by fluorescence and the angle correction during sectioning (S2 Fig). In this way, the section obtained for scanning electron microscopy (SEM) and nanoSIMS could be matched to the 3D fluorescence image with a high degree of accuracy (S2 Fig). The sections were then imaged by SEM (Fig 1D), and the same section was then coated with 5-nm gold and transferred for nanoSIMS analysis. BDQ contains a bromine atom, so we determined its localisation by the intensity of the ^79^Br ion signal [10]. The regions imaged by SEM were identified using the optical microscope in the nanoSIMS. The sample was scanned with a focused ^133^Cs^+^ and secondary ions (^12^C^–^, ^12^C^14^N^–^, ^79^Br^–^, ^32^S^–^, and ^31^P^–^), and secondary electrons were collected (Fig 1E). The ^12^C^14^N^–^ and ^31^P^–^ signals were useful to show the morphology of cells and tissues, with ^12^C^14^N^–^ signals largely from proteins and the highest ^31^P^–^ signals are from nucleic acids and structures we believe are polyphosphates in Mtb.

To correlate across imaging modalities with subcellular resolution, endogenous structures were used as landmarks. LD were located by fluorescent staining in the optical image, ultrastructure in the SEM image, and ^32^S^–^ signal in the ion image. The ^32^S^–^ signal was due to the osmium/thiocarbohydrazide staining of lipids. Bacteria were localised by fluorescence (E2-Crimson signal), ultrastructure, and ^31^P^–^ signal in the ion image. The cell nucleus was aligned using ultrastructure and the ^31^P^–^ signal (Fig 1F). Concurrent with previous CLEIM in vitro studies, we found that BDQ accumulated heterogeneously in LD and Mtb, with particularly high levels in infected foamy macrophages (Fig 1F and S3 Fig). Importantly, some bacteria contained high levels of the antibiotic, whereas others did not show any signal, indicating that the antibiotic is not able to evenly reach throughout intracellular bacteria present in the infected tissue (Fig 1F and S3 Fig).

**Fig 2:**
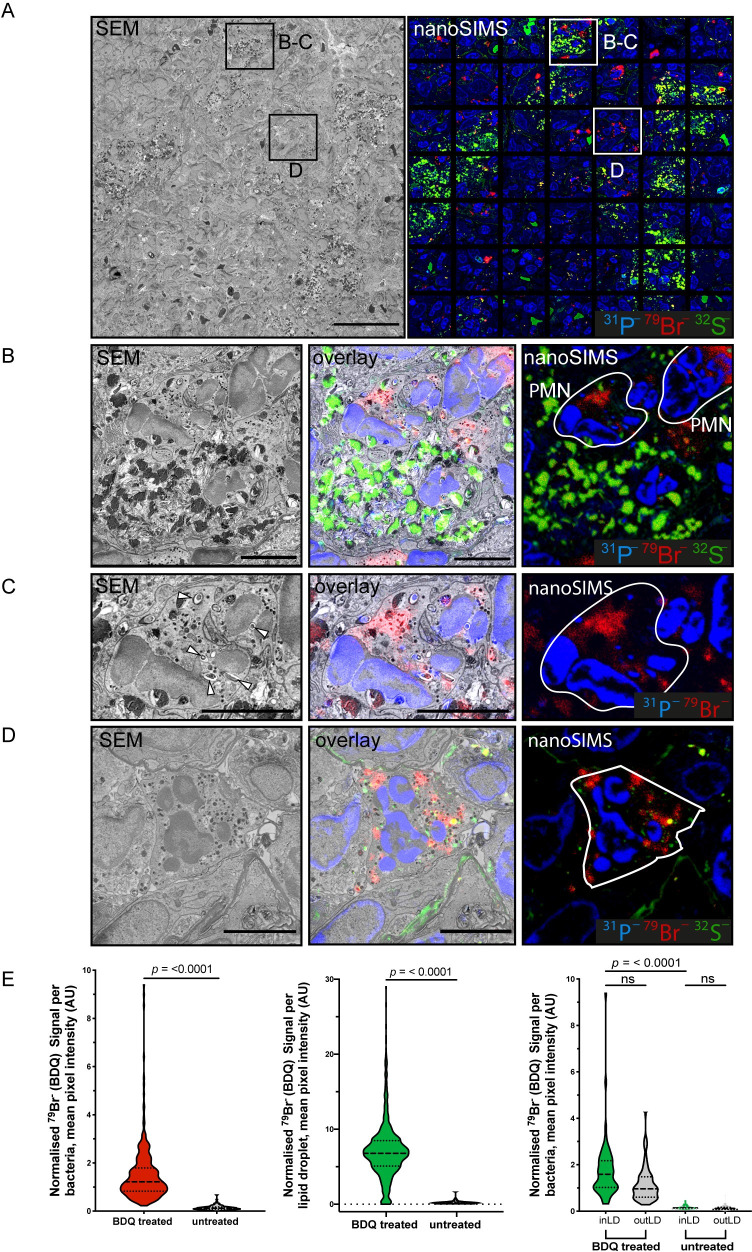
Quantitative distribution of BDQ reveals intracellular distribution of BDQ in foamy macrophages and PMN within granulomatous lesions. (**A**) Left: a tiled SEM image of a region of granulomatous lung tissue indicating the zoomed areas. Scale bar = 40 μm. Right: a mosaic of 49 individual ion micrographs showing the quantitative distribution of ion signals in the respective area of tissue. Sulphur ^32^S^–^ is shown in green, bromine ^79^Br^−^in red, and phosphorus ^31^P^–^ in blue. (**B**) PMN cells are recruited to the foamy macrophage rich lesions. SEM of zoom 1 (left) and the distribution of secondary ions ^31^P^–^, ^79^Br^–^, and ^32^S^–^ (right). Overlaid image between SEM (left) and secondary ion (right) is shown in the center. PMN are demarcated by the white boundary line. Scale bar = 5 μm. (**C**) An infected PMN from panel B showing strong accumulation of BDQ. Overlay between the SEM and secondary ion signals for ^79^Br^−^(red) and ^31^P^–^ (blue) is depicted in the center. White arrowheads indicate intracellular bacteria. Scale bar = 5 μm. (**D**) BDQ strongly enriched in PMN that are not only associated with the foamy macrophage-enriched areas. SEM of zoom 2 (left) and the distribution of secondary ions ^31^P^–^, ^79^Br^–^, and ^32^S^–^ (right). Overlaid image of SEM and secondary ions is depicted at the center. Demarcation of PMN is outlined by the white boundary line. Scale bar = 5 μm. (**E**) Left panel: quantitative analysis of BDQ associated with Mtb in BDQ treated and untreated mice. Center panel: quantitative analysis of BDQ associated with LD in BDQ treated and untreated mice. Right panel: BDQ association to Mtb inside or outside LD in untreated vs BDQ-treated mice. Data show mean ± standard deviation. *t* test adjusted for multiple comparisons. ns, nonsignificant; *p*-value is as shown. At least 140 objects were counted from each treatment condition. Data can be found in S1 Data. AU, arbitrary units; BDQ, bedaquiline; LD, lipid droplet; nanoSIMS, nanoscale secondary ion mass spectrometry; PMN, polymorphonuclear; SEM, scanning electron microscopy.

Taking advantage of this method, we then focused on a more quantitative approach (S4 Fig) to analyse intracellular antibiotic localisation in the lung lesions. For that, we performed a combined tile scanning by SEM and nanoSIMS covering larger areas of the tissue (Fig 2A). This allowed to define the distribution of BDQ in single cells and bacteria (S2 Movie). Unexpectedly, we found that BDQ not only localised in lipophilic environments (e.g., in LD) but also in non-lipophilic cellular environments. Specifically, we found that BDQ strongly accumulated in polymorphonuclear (PMN) cells. Antibiotic-rich PMN were present both alongside (Fig 2B and 2C) and away from areas enriched with foamy macrophages (Fig 2D). In contrast to macrophages where the ^79^Br^−^signal was primarily associated with LD, in PMN, the ^79^Br^−^signal was not only associated with granules, small LDs (similarly to macrophages) or eventually the cytosol (S5 Fig). Thus, in tissues, BDQ accumulates in a cell type–dependent manner across at least 2 Mtb-infected cell populations (foamy macrophages and PMN) with very different metabolic and functional properties, which is an important aspect to consider in the context of intracellular accumulation of antibiotics. Confirming our previous observations, quantitative analysis revealed that BDQ heterogeneously accumulated in LD and Mtb (Fig 2E and S1 Data). Both Mtb outside and inside LD accumulated BDQ (Fig 2E and S1 Data). These PMN cells are likely neutrophils recruited to the granuloma as reported in this mouse model of tuberculosis (TB) infection [14]. Neutrophils are rich in myeloperoxidase (MPO), a peroxidase that produces hypochlorous acid from hydrogen peroxide and chloride anion or hypobromous acid if bromide anion is present [15]. However, in untreated mice, the ^79^Br^−^signal was significantly lower and only slightly associated with PMN granules, indicating that the ^79^Br^−^signal was primarily coming from the antibiotic (S5 Fig and S3 Data). The ^79^Br^–^/^12^C^14^N^–^ were used when comparing the BDQ-treated and non-treated tissues. The normalisation to ^12^C^14^N^–^ was to compensate possible minor variations in the primary ion current during imaging.

### Discussion

Altogether, we report the development of a correlative approach in tissue to define the subcellular localisation of antibiotics in infected cells within tissues. This multimodal imaging approach represents a powerful methodological advance to investigate if drugs reach their intracellular targets. Using this approach, we identified that in the lungs of *M*. *tuberculosis*–infected mice, the antibiotic BDQ heterogeneously localised to intracellular bacteria and LD of foamy macrophages. We also found that BDQ significantly accumulated in specific cell types such as PMN, likely neutrophils, recruited into granulomatous lesions. Therefore, CLEIMiT enabled us to characterise the antibiotic distribution across multiple cell types, revealing another niche of drug accumulation in lungs. Of note, BDQ has a very long half-life and only reaches steady state after several weeks of treatment, during which time it will continue to accumulate in tissues and cells. Thus, the drug distribution we observe here will likely change with time until steady state is reached in tissues. Further studies using CLEIMiT will allow to investigate how BDQ distribution changes during treatment.

CLEIMiT is readily applicable to other drugs and not only for antibiotics or bromine-containing drugs but also any drugs detectable by nanoSIMS. Ion microscopy methods using nanoSIMS represents a good combination of spatial resolution and sensitivity to map drugs that contain elements other than bromine that are low in the biological systems such as platinum [16], gold [17], and iodine [18]. What makes the approach more widely applicable is the nanoSIMS capability to detect stable isotope-labelled molecules with high resolution such as molecules that are labelled with ^2^H [18,19], ^13^C [18,20], and ^15^N [21,22]. All of these stable isotopes can be used to label drugs of interest and mapped using the multiplexed imaging potential of CLEIMiT. This correlative approach is in principle compatible with transmission electron microscopy (TEM), which will significantly improve the resolution of the EM modality, but with limitations. First, the nanoSIMS is a destructive technique, and the ideal sample is a semi-thin flat section (>300 nm) with a solid support. The nanoSIMS image quality is potentially compromised for imaging on thin sections (e.g., <200 nm) mounted on TEM grids. Second, the quantification of secondary ions on thin sections may not be as reliable as the pre-imaging implantation of ^133^Cs^+^ restricted to a low dose on thin sections. Third, thin sections mounted on the TEM grids may not allow nanoSIMS imaging on areas of hundreds of microns by hundreds of microns as they are not as stable as semi-thin sections mounted on silicon wafers or other conductive flat surfaces.

Importantly, in this study, we used a physiologically relevant treatment dose of BDQ. The detection limit of each elements or isotopes are different with nanoSIMS, and they are still not well documented in biological systems. However, multiple studies have demonstrated its capability to map drugs and other molecules with high resolution and sensitivity. From a pharmacokinetics/pharmacodynamics point of view, it would be important to define where and when the PMN internalise the antibiotic, since these cells are motile and actively recruited during lung inflammation. CLEIMiT also opens the possibility to define if other antibiotics currently used in the clinic are able to penetrate intracellular environments containing bacteria. We have used mainly cellular ultrastructure and morphology to identify foamy macrophages and PMN, acknowledging that this could be a limiting step of the method described here to undoubtedly define cell types. In this context, the combination of CLEIMiT with transgenic mice expressing specific fluorescent markers of cellular subtypes (e.g., myelocytic, endothelial, epithelial, etc.) will provide a suitable experimental setting to define in which cells antibiotics preferentially distribute and/or accumulate.

## Materials and methods

### Murine aerosol *M*. *tuberculosis* infection

*M*. *tuberculosis* H37Rv wild type (WT) was kindly provided by Douglas Young (The Francis Crick Institute, United Kingdom). E2Crimson-Mtb was generated by transformation with pTEC19, a gift from Lalita Ramakrishnan (Addgene 30178). Bacteria were verified by sequencing and tested for the virulence-related lipids phthiocerol dimycocerosates (PDIM) positivity. C3HeB/FeJ mice were bred under pathogen-free conditions at The Francis Crick Institute. Animal studies and breeding were approved by The Francis Crick Institute (London, UK) ethical committee and performed under UK Home Office project license PPL 70/8045. Infections were performed in the category 3 animal facility at The Francis Crick Institute. For aerosol infection, *M*. *tuberculosis* expressing E2crimson were grown to mid log-phase (OD_600_ of 0.6) in 7H9 (Sigma-Aldrich, Germany M0178) supplemented with 10% albumin dextrose-catalase (BD Biosciences, United States of America 212351) and 0.05% TWEEN-80 (Sigma-Aldrich, Germany P1754). An infection sample was prepared from this, to enable delivery of approximately 100 CFU/mouse lung using a modified Glas-Col aerosol infection system.

### Treatment with bedaquiline

Three weeks after infection the treatment group was given 25 mg/kg of BDQ (dissolved in 2-hydroxypropyl-β-cyclodextrin) (MedChemExpress, USA HY-14881) daily for 5 days via oral gavage, while the control group were given only 2-hydroxypropyl-β-cyclodextrin. At the end of treatment, mice were humanely killed by anaesthesia, then the lungs were either perfused with 10% neutral buffered formalin and excised (S1A Fig) or bacterial counts were determined by plating serial dilutions of homogenates on duplicate Middlebrook 7H11 (Sigma-Aldrich, M0428) containing OADC (BD Biosciences, 212240). Colonies were counted 2 to 3 weeks after incubation at 37°C. The data at each time point are the means of 5 mice/group +/–SEM (S1B Fig and S2 Data). CFU/lung are calculated from the average of the duplicate multiplied by the volume of the dilution and the sample volume.

### CLEIMiT (Correlative light, electron, and ion microscopy in tissue)

#### Micro-computed tomography (μCT)

μCT imaging of whole lung: Whole lungs were incubated overnight at room temperature in 25% isotonic Lugol’s solution w/v for contrast then in 0.75% low melting point agarose (LMA, 16520050, Thermo Fisher Scientific, USA) w/v in 200 mM HEPEs incubated at 37°C for 1 hour. Lungs were then set in 4% LMA and imaged using a Xradia 510 Versa 3D X-ray microscopes (Zeiss, Germany) with the following acquisition setting: 0.4× objective, pixel size = 7 μm, pixel binning of 2, source filter = LE1, Voltage = 40 kV, Wattage = 3.0 W. Tomogram reconstruction was carried out using the Zeiss Scout and Scan Software (Zeiss, Germany). Visualisation and fine measurements were taken from a 3D volume reconstruction using Zeiss XM3D viewer software (Zeiss, Germany).

μCT imaging of resin-embedded tissue: A tissue slice was imaged using a Xradia 510 Versa 3D X-ray microscopes (Zeiss, Germany) with the following acquisition settings: 4× objective, pixel size = 2.8 μm, pixel binning of 2, source filter = LE2, Voltage = 40 kV, Wattage = 3.0 W. Tomogram reconstruction was carried out using the Zeiss Scout and Scan Software (Zeiss, Germany). Visualisation and fine measurements were taken from a 3D volume reconstruction using Zeiss’ XM3D viewer software (Zeiss, Germany). Moreover, 3D measurements of the resin section were used to give precise co-ordinates for the location of the fluorescently imaged area in the resin block (S2B Fig) and to determine the precise angle of advance for the diamond knife when trimming the resin block.

#### Vibratome sections

The lungs were separated into the 4 constituent lobes of the right lung (superior, middle, inferior, and postcaval) and left lung. Lobes were then embedded separately in 4% w/v LMA in 200 mM HEPES in agarose moulds (Sigma-Aldrich, Germany E6032-1CS). The mould was placed on ice to cool and harden for sectioning. Moreover, 100-μm sections were cut using a VT1200S fully automated vibrating blade microtome (Leica Biosystems, Germany). Upon calibration of the instrument, sections were cut at a cutting speed of 0.35 mm/sec and an amplitude of 1.0 mm. Individual sections were sequentially removed and collected in a pre-labelled 12-well plate containing 1,000 μl of 200 mM HEPEs buffer.

#### Fluorescence staining and imaging of mouse lung sections

In a 24-well plate, 100-μm lung slices were washed twice in 200 mM HEPES buffer then incubated for 20 minutes in a staining solution containing 0.715 μM DAPI (4′,6-diamidino-2-phenylindole) (Thermo Fisher Scientific D1306) and 10 mg/L BODIPY 493/503 (4,4-difluoro-1,3,5,7,8-pentamethyl-4-bora-3a,4a-diaza-s-indacene) (Invitrogen, UK D3922) in 200 mM HEPES buffer. Slices were washed twice with 200 mM HEPES and transferred to a glass slide and positioned to lie flat, unfolded across the surface. Excess buffer was removed, along with any remaining agarose and 10-μL DAKO fluorescent mounting medium (Agilent, UK S3023) added. A cover glass (NA = 1.5) was gently placed upon the tissue and the medium allowed to set. An inverted Leica TCS SP8 microscope running LAS X acquisition software with Navigator module (Leica Microsystems, Germany), equipped with 405 nm, Argon laser, 561 nm, 633 nm, and HyD detectors was used to image the tissue fluorescence with the following Lasers: 405nm (DAPI), 488nm (BODIPY), and 561nm (Mtb-E2Crimson). In the first instance, the entire tissue section was imaged with a tile scan using the 10× objective lens. Regions of interest (ROI) were then identified based upon areas of tissue showing high degrees of cellular infiltration, indicated by DAPI staining, and the accumulation of highly lipid foamy cells, indicated by BODIPY staining. Selected ROI were then imaged at higher resolution using a 40× oil objective and z-stack. Voxel size was adjusted to half the theoretical limit of the lens in x and y and 0.5 μm in z. Fields of view were chosen to include cellular architecture such as airway passages as well as erythrocytes and vessels of the circulatory system, which appear as open space in the tissue, and can later be used as landmarks to help locate the ROI in downstream correlation. After imaging, slides were submerged in 200 mM HEPES and incubated at 4°C until the mounting medium dissolved and the tissue was released. Slices were then stored in 1.25% glutaraldehyde (Sigma-Aldrich, Germany G5882), in 200 mM HEPES (Sigma-Aldrich H0887), pH 7.4 until embedding.

#### Resin embedding

Fluorescently imaged slices were processed for SEM and nanoSIMS in a Biowave Pro (Pelco, USA) with use of microwave energy and vacuum. Samples (approximately 0.3 to 0.4 mm^3^) were twice washed in HEPES (Sigma-Aldrich H0887) at 250 W for 40 seconds, postfixed using a mixture of 2% osmium tetroxide (Taab O011) 1.5% potassium ferricyanide (Taab, P018) (v/v) at equal ratio for 14 minutes at 100 W power (with/without vacuum 20 Hg at 2-minute intervals). Samples were washed with distilled water twice on the bench and twice in the Biowave 250 W for 40 seconds, 1% thiocarbohydrazide (Sigma-Aldrich 223220) in distilled water (v/v) for 14 minutes at 100 W power (with/without vacuum 20 Hg at 2-minute intervals), washing cycle was repeated as before, then incubated with 2% osmium tetroxide (Taab, O011) distilled water (w/v) for 14 minutes at 100 W power (with/without vacuum 20 Hg at 2-minute intervals). Samples were washed as before. Samples were stained with 1% aqueous uranyl acetate (Agar Scientific, UK AGR1260A) in distilled water (w/v) for 14 minutes at 100 W power (with/without vacuum 20 Hg at 2-minute intervals) then washed using the same settings as before. Samples were dehydrated using a step-wise ethanol series of 50, 75, 90, and 100%, then washed 4 times in absolute acetone at 250 W for 40 seconds per step. Samples were infiltrated with a dilution series of 25, 50, 75, and 100% Durcupan ACM (Sigma-Aldrich 44610) (v/v) resin to acetone. Each step was for 3 minutes at 250 W power (with/without vacuum 20 Hg at 30-second intervals). Samples were then cured for a minimum of 48 hours at 60°C.

#### Resin block trimming

Referring to measurements from the 3D volume reconstruction, generated by μCT, the sample block was trimmed, coarsely by a razor blade then finely trimmed using a 35 degrees ultrasonic, oscillating diamond knife (DiATOME, Switzerland) set at a cutting speed of 0.6 mm/s, a frequency set by automatic mode, and a voltage of 6.0 V, on a ultramicrotome EM UC7 (Leica Microsystems, Germany) to remove all excess resin and tissue surrounding the ROI. Precise measurements, derived from the μCT reconstruction, were used to further cut into the tissue, to the depth corresponding with the fluorescent area previously imaged.

### Nanoscale secondary ion mass spectrometry (nanoSIMS)

The sections were imaged by SEM and nanoSIMS as previously described [10]. A total of 500-nm sections were cut using ultramicrotome EM UC7 (Leica Microsystems, Germany) and mounted on 7 mm by 7 mm silicon wafers. Sections on silicon wafers were imaged using a FEI Verios SEM (Thermo Fisher Scientific) with a 1 kV beam with the current at 200 pA. The same sections were then coated with 5-nm gold and transferred to a nanoSIMS 50L instrument (CAMECA, France). The regions that were imaged by SEM were identified using the optical microscope in the nanoSIMS. A focused ^133^Cs^+^ beam was used as the primary ion beam to bombard the sample; secondary ions (^12^C^–^, ^12^C^14^N^–^, ^79^Br^–^, ^32^S^–^, and ^31^P^–^) and secondary electrons were collected. A high primary beam current of approximately 1.2 nA was used to scan the sections to remove the gold coating and implant ^133^Cs^+^ to reach a dose of 1 × 10^17^ ions/cm^2^ at the steady state of secondary ions collected. Identified ROIs were imaged with an approximately 3.5 pA beam current and a total dwell time of 10 ms/pixel. Scans of 512 × 512 pixels were obtained.

#### Image alignment

Tissue derived micrograph and nanoSIMS/micrograph correlation: Ion and fluorescent images were aligned to EM micrographs with Icy 2.0.3.0 software (Institut Pasteur, France), using the ec-CLEM Version 1.0.1.5 plugin. No less than 10 independent fiducials were chosen per alignment for 2D image registration. When the fiducial registration error was greater than the predicted registration error, a nonrigid transformation (a nonlinear transformation based on spline interpolation, after an initial rigid transformation) was applied as previously described [23].

#### Quantification and statistical analysis

Ion quantification: Secondary ion signal intensities were quantified in ImageJ with the OpenMIMS v3.0.5 plugin.

Quantification of BDQ within bacteria: Bacteria (_total_n = 472) were manually outlined with the assistance of SEM images and the ^31^P^–^ signal. Ratio values (^79^Br^–^/^12^C^14^N^–^) for bacteria were divided by the area of their respective ROI to give mean normalised pixel intensity in arbitrary units (AU) for each condition. Mean normalised pixel intensity in AU per ROI was plotted against condition using GraphPad Prism 8 software (USA), and 2-tailed *p*-value was determined by an unpaired, nonparametric Mann–Whitney U test to assess statistical significance.

Quantification of BDQ in lipid droplets: LDs (_total_n = 1404) were outlined using the (^32^S^–^/1) ratio value. The resulting ratio image was summed and processed with a Gaussian blur filter (sigma radius = 2 pixels). A threshold was applied to mask the image. ROIs were identified by particle analysis and verified by comparison with the respective SEM image. Masked areas were overlaid to the ^79^Br^–^/^12^C^14^N^–^ ratio image of the same area of tissue. ROIs with less than 5 pixels in size were excluded from the analysis. Mean normalised pixel intensity in AU per ROI was plotted against condition using GraphPad Prism 8 software. Two-tailed *p*-value was determined by an unpaired, nonparametric Mann–Whitney U test to assess statistical significance.

Quantification of BDQ in bacteria inside LD: Bacteria (_total_n = 282) were manually outlined, and localisation defined to be either inside lipid droplets (inLD) or outside lipid droplets (outLD) with the assistance of SEM images and the ^31^P^–^ and ^32^S^–^ signal. Ratio values (^79^Br^–^/^12^C^14^N^–^) for bacteria were divided by the area of their respective ROI to give mean normalised pixel intensity in AU for each condition. Two-tailed *p*-values were determined by an unpaired, nonparametric Kruskal–Wallis test with Dunn’s correction to assess statistical significance.

Quantification of BDQ in PMN and foamy cells: PMN cells (*n* = 22) and foamy cells (*n* = 76) were manually outlined based on distinct morphological features using scanning electron micrographs. Continuous osmic stained plasma membranes delineating the cell boundary and compared with the ^12^C^14^N^–^ and ^32^S^–^ signal to define the cell edge. Ratio values (^79^Br^–^/^12^C^14^N^–^) for PMN were divided by the area of their respective ROI to give mean normalised pixel intensity in AU for each condition. Mean normalised pixel intensity in AU per ROI was plotted against condition using GraphPad Prism 8 software. Two-tailed *p*-value was determined by an unpaired, nonparametric Mann–Whitney U test to access statistical significance.

## Supporting information

S1 Fig Overview of the infection model and treatment. (A) Diagram of the infection and treatment experimental setting. (B) CFUs in the lungs of mice at day 0 of infection (inoculum) and treated with either BDQ or vehicle. Data can be found in S2 Data. (C) μCT of whole lung showing granulomatous lesions. BDQ, bedaquiline; CFU, colony-forming unit; μCT, micro-computed tomography.
(PDF)

S2 Fig Strategy for correlative light (fluorescence), electron (SEM), and ion (nanoSIMS) microscopy. (A) Diagram of the sectioning strategy for correlation, including the different imaging modalities and depth of imaging. A^o^ represents (arrow) the need to calculate and adjust the angle of incidence of the diamond knife with the resin block so as to achieve parallel sectioning from the surface of the tissue, through the entire block. Imaging modalities are applied to the referred sections, at different depths as shown. (B) Correlation between fluorescent ROI and μCT of resin-embedded section and positioning localisation of the SEM section for correlation between nanoSIMS/SEM and fluorescence. Middle panels show different orthogonal slices of the 3D section used to localise area and calculate angle and depth for further sectioning. The intersecting lines indicate the precise location of the target cell within the resin-embedded section. Distances which are used to calculate angles are shown in blue numbers. These localisations were used to zoom in the ROI (zoom) and obtain the SEM image corresponding to the fluorescent image in the upper panel. nanoSIMS, nanoscale secondary ion mass spectrometry; ROI, regions of interest; SEM, scanning electron microscopy; μCT, micro-computed tomography.
(PDF)

S3 Fig BDQ accumulates heterogeneously in LD and Mtb, with particularly high levels in infected foamy macrophages. (A) Fluorescent microscopy of an ROI as in Fig 1: (i) cellular infiltration and the accumulation of BODIPY (green) positive cells. Cells infected with Mtb-E2Crimson (red) are also visible throughout this region. Scale bar = 100 μm. (ii) Zoomed in image showing the selected foamy cell infected with Mtb-E2Crimson (red) from (i) for correlative analysis. Scale bar = 15 μm. Lower panels show the ion microscopy for the selected cell including ^12^C^14^N^–^, ^31^P^–^, ^79^Br^–^, and ^32^S^–^. Compass indicates the orientations of secondary ion images with regard to the fluorescent image above. (B) CLEIMiT: right, a correlated image overlaying the ^79^Br^−^and ^31^P^–^ signals with the SEM image. Center, the corresponding SEM image of the infected foamy cell. Left, a correlated image overlaying fluorescent signal from BODIPY (green) and Mtb-E2Crimson (red) against the SEM image. Scale bar = 5 μm. (B.i) An example of Mtb demonstrating lower ^79^Br^−^signal (0.507). The panels show the SEM image of ROI B.i (left). An ion micrograph for area (B.i) shows the distribution of ^79^Br^−^signal and bacteria are indicated by a white boundary (center). The final panel image shows an overlaying ion (^31^P^–^, ^79^Br^–^) and SEM micrograph (right). Scale bar = 2 μm. (B.ii) An example of Mtb demonstrating higher ^79^Br^−^signal (1.595). The panel shows the SEM image of ROI B.ii (left). An ion micrograph for area (B.ii) shows the distribution of ^79^Br^−^signal and bacteria are indicated by a white boundary (center). The final panel image shows an overlaying ion (^31^P^–^, ^79^Br^–^) and SEM micrograph (right). Scale bar = 2 μm. BDQ, bedaquiline; CLEIMiT, correlative light, electron, and ion microscopy in tissue; LD, lipid droplet; ROI, region of interest; SEM, scanning electron microscopy.
(PDF)

S4 Fig Quantitative analysis workflow of BDQ levels in Mtb and LD in foamy macrophages. (A) Masking of Mtb profiles aided by the combination of the SEM profiles and ^31^P^–^ signal and measurement of the ^79^Br^−^signal associated with Mtb (see Materials and methods). Scale bar = 5 μm. (B) Masking of LD profiles aided by the combination of the SEM profiles and ^32^S^–^ signal and measurement of the ^79^Br^−^signal associated with LD (see Materials and methods). Scale bar = 5 μm. BDQ, bedaquiline; LD, lipid droplet; SEM, scanning electron microscopy.
(PDF)

S5 Fig Bromine signal from PMN is primarily associated with BDQ. (A) Representative SEM/nanoSIMS correlated images of PMN in granulomatous lesions in lungs of mice treated with vehicle (untreated) and BDQ (BDQ treated). Scale bar = 5 μm. (B) Quantitative analysis of ^79^Br^−^signal in PMN and foamy cells in lungs of mice treated with vehicle (untreated) and BDQ (treated). Data show mean ± standard deviation. *t* test adjusted for multiple comparisons. ns, nonsignificant; *p*-value is as shown. A total of 22 PMN and 76 foamy cells were counted. Data can be found in S3 Data. BDQ, bedaquiline; nanoSIMS, nanoscale secondary ion mass spectrometry; PMN, polymorphonuclear; SEM, scanning electron microscopy.
(PDF)

S1 Data Quantitative analysis of ^79^Br^−^signal associated with LDs and bacteria in cells in lungs of mice treated with vehicle (untreated) and BDQ (BDQ treated). BDQ, bedaquiline; LD, lipid droplet.
(XLSX)

S2 Data CFU in the lungs of mice at day 0 of infection (inoculum) and treated with either BDQ or vehicle. BDQ, bedaquiline; CFU, colony-forming unit.
(XLSX)

S3 Data Quantitative analysis of ^79^Br^−^signal in PMN and foamy cells in lungs of mice treated with vehicle (untreated) and BDQ (treated). BDQ, bedaquiline; PMN, polymorphonuclear; μCT, micro-computed tomography.
(XLSX)

S1 Movie μCT of infected lungs showing lesions. (MP4)

S2 Movie Ion images and intracellular localisation of BDQ in different cell types within granulomatous lesions. BDQ, bedaquiline; μCT, micro-computed tomography.
(MP4)

## Abbreviations

AU: arbitrary units
BDQ: bedaquiline
CFU: colony-forming units
CLEIM: correlative light, electron, and ion microscopy
CLEIMiT: correlative light, electron, and ion microscopy in tissue
LD: lipid droplet
LMA: low melting point agarose
MALDI-MSI: matrix-assisted laser desorption–ionisation mass spectrometric imaging
MPO: myeloperoxidase
nanoSIMS: nanoscale secondary ion mass spectrometry
PDIM: phthiocerol dimycocerosates
PMN: polymorphonuclear
ROI: regions of interest
SEM: scanning electron microscopy
TB: tuberculosis
TEM: transmission electron microscopy
μCT: micro-computed tomography
WT: wild type
