# Supplementary figures and images for: Correlative light electron ion microscopy reveals in vivo localisation of bedaquiline in Mycobacterium tuberculosis–infected lungs
Source: PLoS Biol. 2020 Dec 31;18(12):e3000879. doi: 10.1371/journal.pbio.3000879 (PMC7810513; doi:10.1371/journal.pbio.3000879)

Figure S1

A

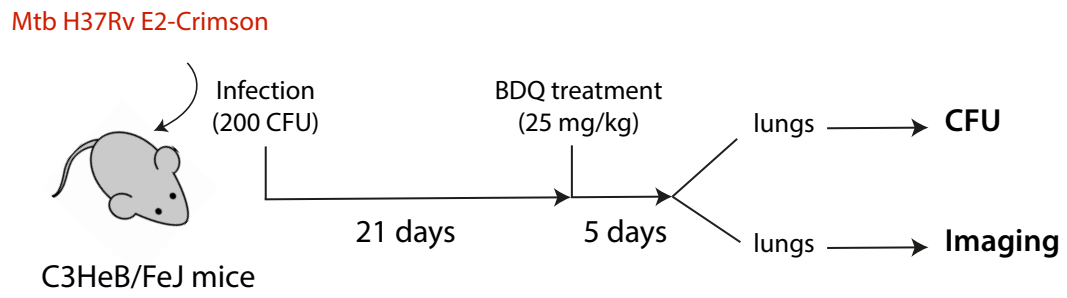

B

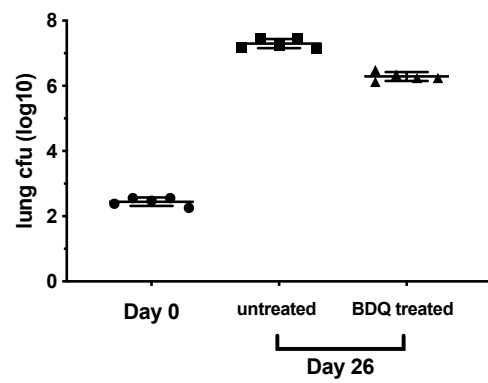

C

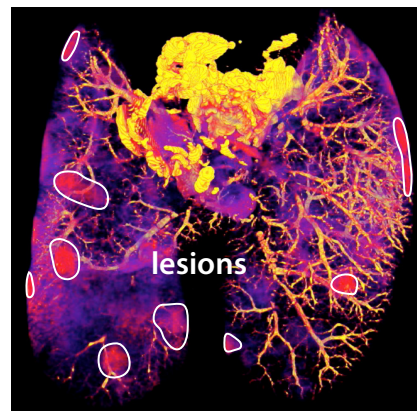

Supplement: S1 Fig — (A) Diagram of the infection and treatment experimental setting. (B) CFUs in the lungs of mice at day 0 of infection (inoculum) and treated with either BDQ or vehicle. Data can be found in S2 Data. (C) μCT of whole lung showing granulomatous lesions. BDQ, bedaquiline; CFU, colony-forming unit; μCT, micro-computed tomography. (PDF) [file pbio.3000879.s001.pdf]

Figure S2

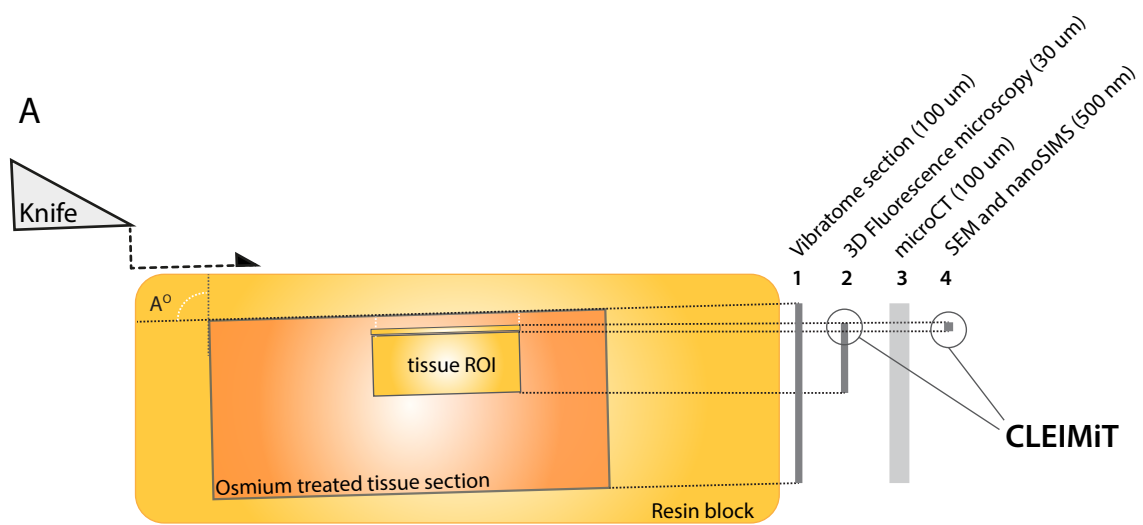

**B**

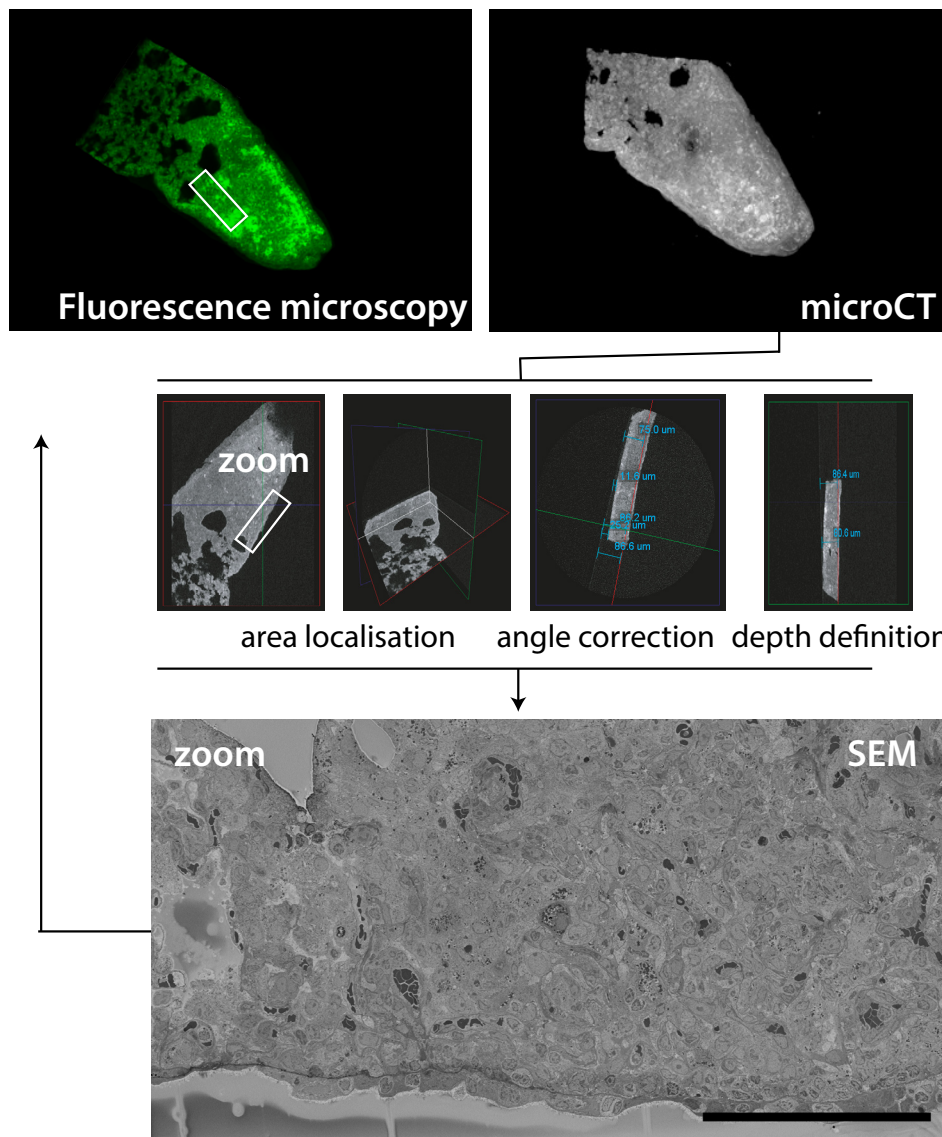

Supplement: S2 Fig — (A) Diagram of the sectioning strategy for correlation, including the different imaging modalities and depth of imaging. Ao represents (arrow) the need to calculate and adjust the angle of incidence of the diamond knife with the resin block so as to achieve parallel sectioning from the surface of the tissue, through the entire block. Imaging modalities are applied to the referred sections, at different depths as shown. (B) Correlation between fluorescent ROI and μCT of resin-embedded section and positioning localisation of the SEM section for correlation between nanoSIMS/SEM and fluorescence. Middle panels show different orthogonal slices of the 3D section used to localise area and calculate angle and depth for further sectioning. The intersecting lines indicate the precise location of the target cell within the resin-embedded section. Distances which are used to calculate angles are shown in blue numbers. These localisations were used to zoom in the ROI (zoom) and obtain the SEM image corresponding to the fluorescent image in the upper panel. nanoSIMS, nanoscale secondary ion mass spectrometry; ROI, regions of interest; SEM, scanning electron microscopy; μCT, micro-computed tomography. (PDF) [file pbio.3000879.s002.pdf]

Figure S3

A

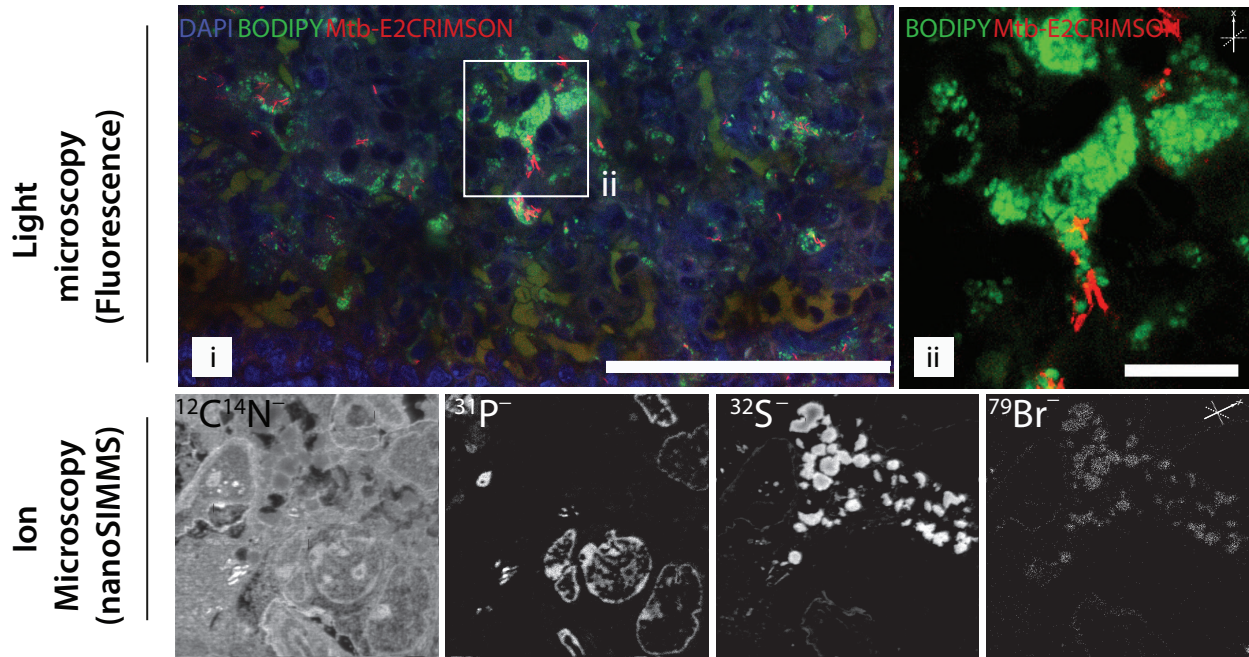

B

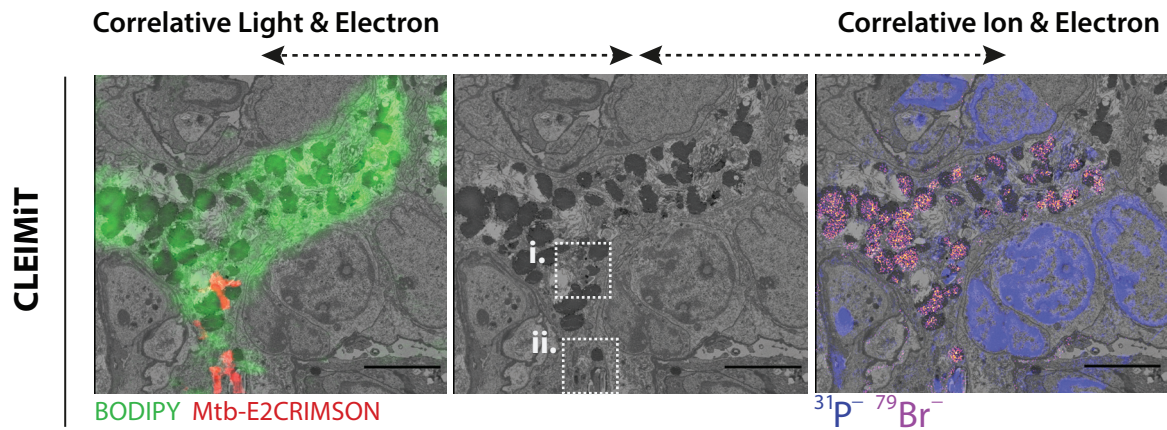

i. low  $^{79}\text{Br}^-$  signal in Mtb

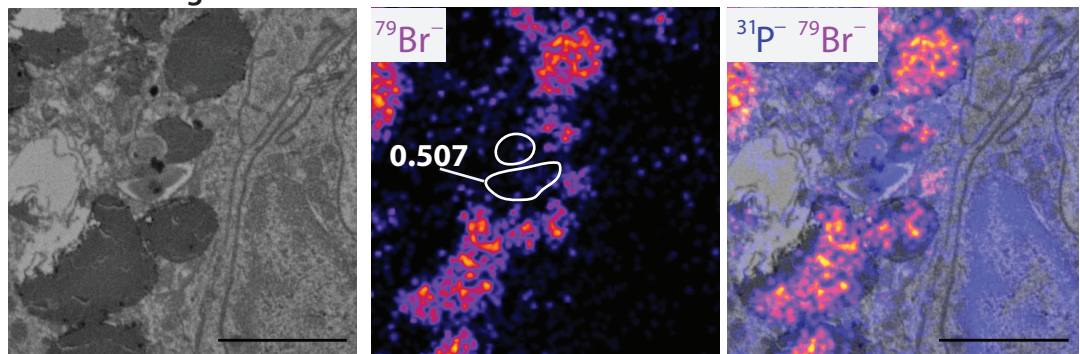

ii. high  $^{79}\text{Br}^-$  signal in Mtb

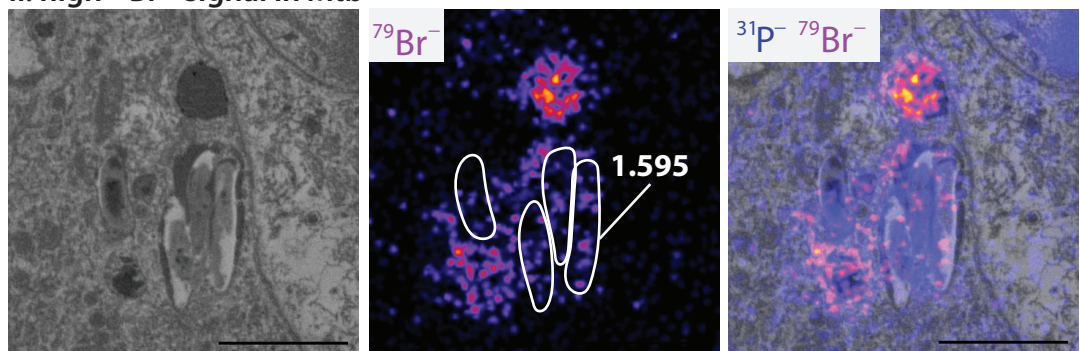

Supplement: S3 Fig — (A) Fluorescent microscopy of an ROI as in Fig 1: (i) cellular infiltration and the accumulation of BODIPY (green) positive cells. Cells infected with Mtb-E2Crimson (red) are also visible throughout this region. Scale bar = 100 μm. (ii) Zoomed in image showing the selected foamy cell infected with Mtb-E2Crimson (red) from (i) for correlative analysis. Scale bar = 15 μm. Lower panels show the ion microscopy for the selected cell including 12C14N–, 31P–, 79Br–, and 32S–. Compass indicates the orientations of secondary ion images with regard to the fluorescent image above. (B) CLEIMiT: right, a correlated image overlaying the 79Br−and 31P– signals with the SEM image. Center, the corresponding SEM image of the infected foamy cell. Left, a correlated image overlaying fluorescent signal from BODIPY (green) and Mtb-E2Crimson (red) against the SEM image. Scale bar = 5 μm. (B.i) An example of Mtb demonstrating lower 79Br−signal (0.507). The panels show the SEM image of ROI B.i (left). An ion micrograph for area (B.i) shows the distribution of 79Br−signal and bacteria are indicated by a white boundary (center). The final panel image shows an overlaying ion (31P–, 79Br–) and SEM micrograph (right). Scale bar = 2 μm. (B.ii) An example of Mtb demonstrating higher 79Br−signal (1.595). The panel shows the SEM image of ROI B.ii (left). An ion micrograph for area (B.ii) shows the distribution of 79Br−signal and bacteria are indicated by a white boundary (center). The final panel image shows an overlaying ion (31P–, 79Br–) and SEM micrograph (right). Scale bar = 2 μm. BDQ, bedaquiline; CLEIMiT, correlative light, electron, and ion microscopy in tissue; LD, lipid droplet; ROI, region of interest; SEM, scanning electron microscopy. (PDF) [file pbio.3000879.s003.pdf]

Figure S4

A Association of BDQ to Mtb

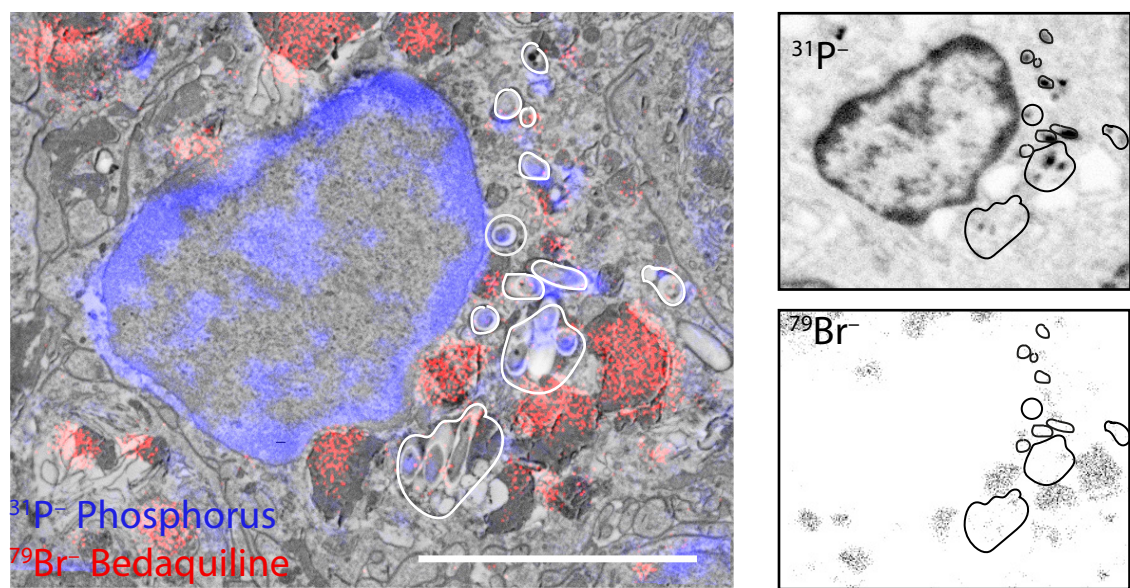

B Association of BDQ to LD

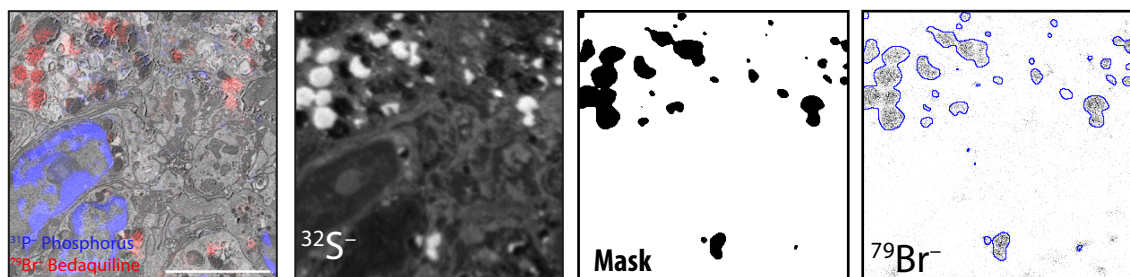

Supplement: S4 Fig — (A) Masking of Mtb profiles aided by the combination of the SEM profiles and 31P– signal and measurement of the 79Br−signal associated with Mtb (see Materials and methods). Scale bar = 5 μm. (B) Masking of LD profiles aided by the combination of the SEM profiles and 32S– signal and measurement of the 79Br−signal associated with LD (see Materials and methods). Scale bar = 5 μm. BDQ, bedaquiline; LD, lipid droplet; SEM, scanning electron microscopy. (PDF) [file pbio.3000879.s004.pdf]

Figure S5

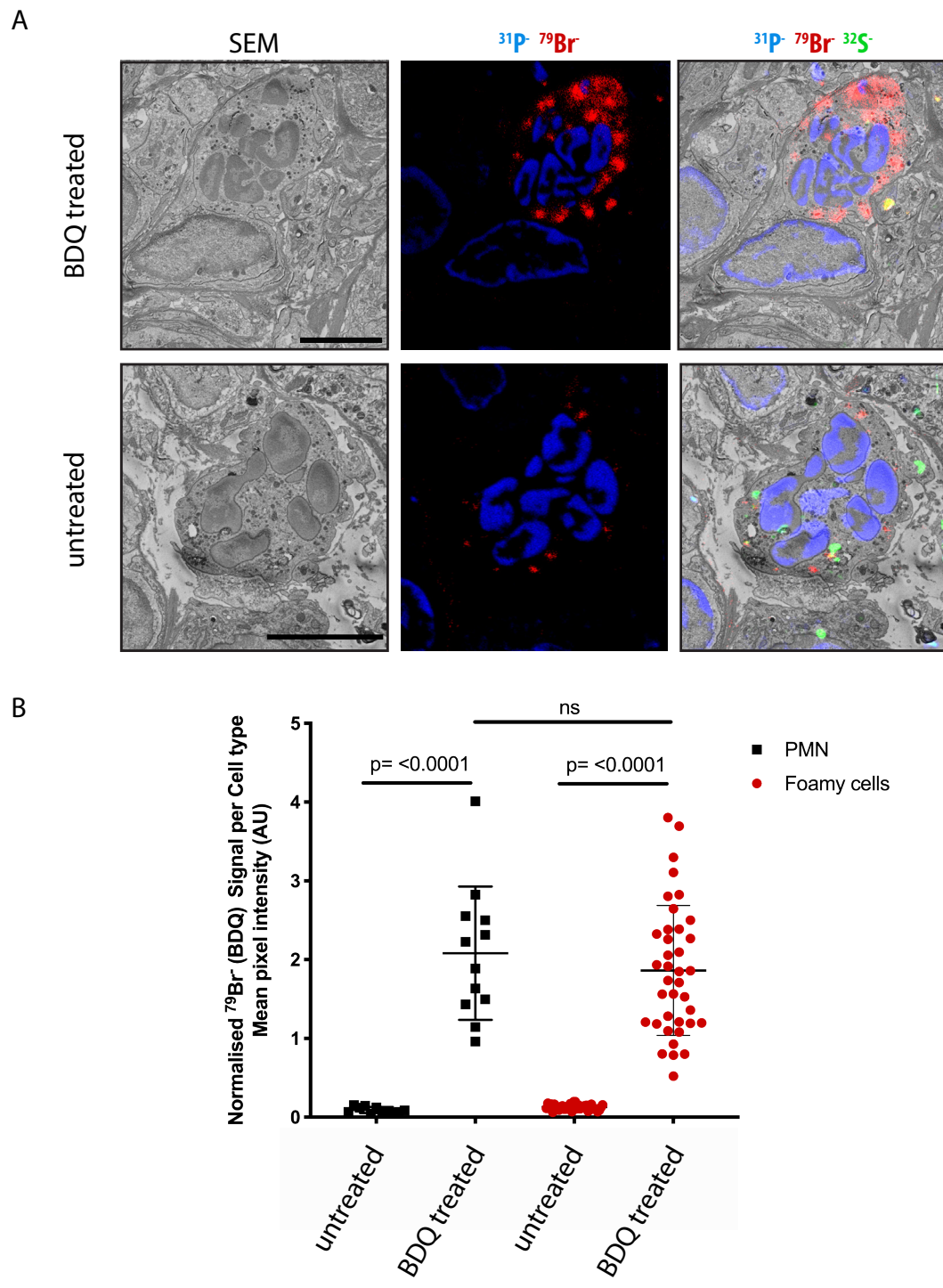

Supplement: S5 Fig — (A) Representative SEM/nanoSIMS correlated images of PMN in granulomatous lesions in lungs of mice treated with vehicle (untreated) and BDQ (BDQ treated). Scale bar = 5 μm. (B) Quantitative analysis of 79Br−signal in PMN and foamy cells in lungs of mice treated with vehicle (untreated) and BDQ (treated). Data show mean ± standard deviation. t test adjusted for multiple comparisons. ns, nonsignificant; p-value is as shown. A total of 22 PMN and 76 foamy cells were counted. Data can be found in S3 Data. BDQ, bedaquiline; nanoSIMS, nanoscale secondary ion mass spectrometry; PMN, polymorphonuclear; SEM, scanning electron microscopy. (PDF) [file pbio.3000879.s005.pdf]
